# Clinical application of repetitive transcranial magnetic stimulation in the treatment of chronic pelvic pain syndrome: a scoping review

**DOI:** 10.3389/fneur.2025.1499133

**Published:** 2025-02-26

**Authors:** Chunmei Luo, Baocheng Zhang, Jing Zhou, Keqiang Yu, Degui Chang

**Affiliations:** ^1^School of Clinical Medicine, Chengdu University of Traditional Chinese Medicine, Chengdu, China; ^2^Anorectal Department, Chengdu Anorectal Hospital, Chengdu, China; ^3^School of Sports Medicine and Health, Chengdu Sport University, Chengdu, China; ^4^TCM Regulation Metabolic Diseases Key Laboratory of Sichuan Province, Hospital of Chengdu University of Traditional Chinese Medicine, Chengdu, Sichuan, China; ^5^Department of Urology, Affiliated Hospital of Chengdu University of Traditional Chinese Medicine, Chengdu, China

**Keywords:** transcranial magnetic stimulation, chronic pelvic pain syndrome, pelvic pain, chronic prostatitis pain, perineal pain, anal and rectal pain, prostatitis

## Abstract

**Introduction:**

Chronic pelvic pain syndrome is a common condition characterized by persistent symptoms that are difficult to treat. Repetitive transcranial magnetic stimulation (rTMS) is considered a safe treatment option for alleviating chronic pelvic pain, but different stimulation protocols can affect pain relief outcomes. Establishing an optimal stimulation protocol can enhance the uniformity and consistency of rTMS to provide a potentially effective therapeutic intervention. This review sought to systematically review and assess the existing literature on transcranial magnetic stimulation in patients experiencing chronic pelvic pain syndrome, evaluate the therapeutic efficacy, and determine the most effective stimulation protocol.

**Methods:**

A comprehensive search was conducted across three databases, supplemented by manual searches. Two researchers independently reviewed and extracted relevant studies and subsequently performed a thorough analysis of all available clinical data.

**Results:**

A total of eight studies were ultimately incorporated into the analysis. These comprised two randomized controlled trials, one self-controlled trial, two case reports, and three prospective studies. All studies demonstrated a notable reduction in pain scores post-treatment.

**Conclusion:**

rTMS has demonstrated efficacy in alleviating pain in individuals suffering from chronic pelvic pain syndrome. It is regarded as a safe intervention with minimal adverse effects. Nonetheless, the variability observed across studies hindered our ability to conclusively determine the most effective stimulation sites and parameters. Additional research is essential to reduce bias, enhance methodological rigor, and ascertain the optimal conditions and indications for brain stimulation to optimize the therapeutic effectiveness of rTMS.

**Systematic Review Registration:**

https://inplasy.com/projects/, identifier INPLASY2023120112.

## 1 Introduction

Chronic pelvic pain syndrome (CPPS) is characterized by enduring or recurrent pain in the pelvic region lasting for at least 3 months without a definitive pathological explanation, and it affects both males and females. The main symptoms include pelvic floor issues, bowel problems, lower urinary tract problems, sexual dysfunction, and gynecological concerns. Moreover, negative emotional, behavioral, and cognitive reactions are often elicited by these symptoms (1). Globally, estimates indicate that the prevalence of CPPS is between 2 and 16% in the male population and up to 24% in the female population (2, 3). CPPS can significantly impact patients’ social engagement and overall quality of life, resulting in heightened feelings of generalized anxiety and depression. As a result, this situation causes great stress for families and society as a whole (4). It is projected that the annual cost of CPPS management will reach $880 million (5).

Individuals with CPPS frequently have impaired pain regulation and increased sensitivity in both the peripheral and central neural systems (6–8). Physical therapy, medication, and nerve block treatments are the current choices for treating CPPS (9, 10). In modern physiotherapy, neuromodulation is being used for the treatment of CPPS patients more and more frequently (11). Transcranial magnetic stimulation (TMS) is a non-invasive, painless neuromodulatory technique that uses pulsed magnetic fields to change the brain metabolism and neural electrical activity. This can result in a range of physiological and biochemical effects (12). Repetitive TMS, or rTMS, is a technique that has been used in the therapeutic management of a variety of neurological and psychiatric conditions since groundbreaking research was published in 1985 that detailed the use of magnetic fields to alter electrical signals within the brain (13–15). Recent developments in neuro-navigation and non-invasive stimulation technology have extended the use of rTMS to the treatment of various types of chronic refractory pain viable (16). Some small-scale studies have revealed that rTMS could be useful in reducing pelvic discomfort in people with CPPS (17, 18). The methodologies employed in the various studies into rTMS have exhibited significant variability, particularly in factors such as the specific rTMS device utilized, the configuration of the coil, and the designated stimulation site, as well as the frequency and intensity of the stimulation. Additionally, differences in the number and duration of stimulations, the total number of sessions conducted, and the overall number of pulses administered have further contributed to this variability. Consequently, it is clear that the outcomes may differ based on the targeted area of stimulation and the selected frequency. At present, there are no standardized protocols for the treatment of CPPS utilizing TMS. The treatment protocols that incorporate TMS for individuals with CPPS remain to be validated through extensive randomized controlled trials. This review seeks to assess the efficacy and safety of TMS in alleviating pain associated with CPPS by examining the current body of literature.

## 2 Methods

### 2.1 Search strategy

To identify studies, we conducted a comprehensive search of the PubMed, Embase, and Cochrane Library databases, utilizing the term “transcranial magnetic stimulation (TMS)” as a medical MESH term, with no time restrictions applied to any of the search fields. The last search date was February 19, 2024. This search encompassed all relevant studies available in these databases. The search was further refined by including one of the following four keywords or MESH terms in the title or abstract: Term A: “Neuralgia*, Perineal,” “Perineal Neuralgia*,” “Pelvic Pain,” “Pelvic Girdle Pain” (*n* = 78); Term B: “Anorectal disease,” “Rect* pain,” “an* pain” (*n* = 49); Term C: “Pelvic Inflammatory Disease,” “Pelvic Infection” (*n* = 15); Term D: “Prostatitis,” “Chronic prostate pain” (*n* = 23). The reference lists of the retrieved articles were manually examined, resulting in the identification of a total of 7 additional articles.

### 2.2 Inclusion and exclusion criteria

#### 2.2.1 Inclusion criteria

The criteria for inclusion were as follows: (1) original research articles; (2) research involving human subjects; (3) publications in the English language; (4) research activities were concentrated on rTMS; and (5) studies evaluating patients showing chronic pelvic pain without any clear underlying pathology.

#### 2.2.2 Exclusion criteria

The exclusion criteria were as follows: (1) book chapters, commentaries, meta-analyses, systematic reviews, letters to the editor, and comments; (2) articles in which rTMS was not employed as a therapy option; (3) articles that did not provide data on the outcomes of pain treatment; and (4) articles involving patients under the age of 18.

The complete set of articles retrieved was used in the final screening, and additional publications meeting the inclusion and exclusion criteria were selected from the reference lists of the retrieved articles.

### 2.3 Synthesis of results

Based on previous literature searches, we anticipated that the included studies would employ diverse therapeutic intervention parameters and exhibit heterogeneity in their dimensions of effect measurement. Due to certain obstacles that prevented us from conducting a thorough and convincing quantitative meta-analysis, we chose to provide a narrative overview of the study results instead (19). All studies were grouped and summarized based on the applied therapeutic parameter models, and the treatment effects were described.

**Figure 1:**
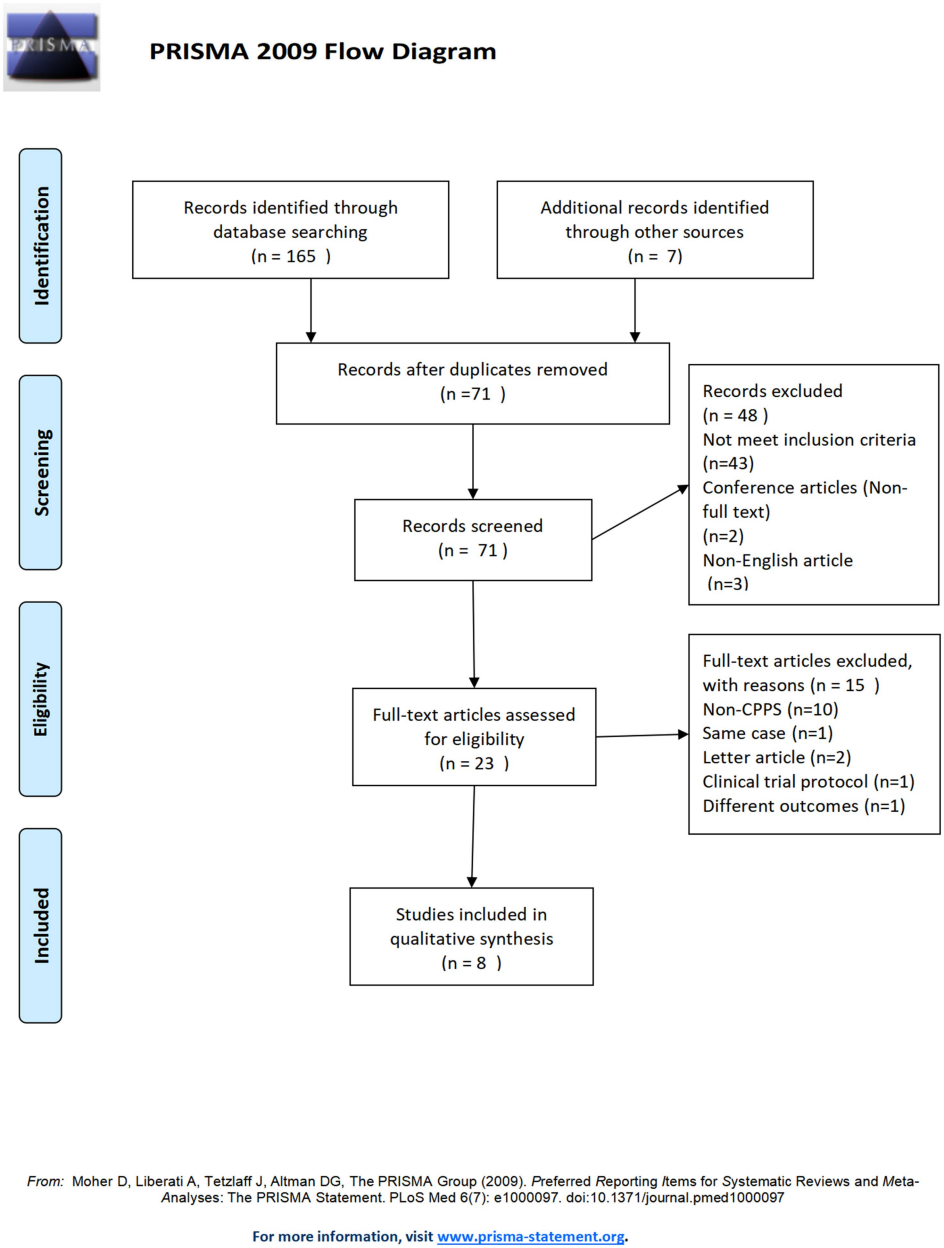
The PRISMA flowchart.

The findings of the literature search and the selected articles are depicted in the PRISMA flowchart presented in Figure 1. Comprehensive articles were ultimately chosen, and further full-text publications that satisfied the inclusion criteria were identified through a review of the references in these articles. Duplicate publications were eliminated by utilizing EndNote software. After that, the titles and abstracts of the articles were scrutinized, with two researchers embarked on a thorough independent review. They carefully selected and kept the full-text versions of all articles that met the inclusion criteria. Patient characteristics, treatment details, and clinical results data were also extracted. Any inconsistencies were addressed through discussions between the two researchers.

### 2.4 Adherence to ethical guidelines

This review was based on previous research and did not involve any studies conducted by the authors on human participants or animals.

## 3 Results

Our literature search strategy identified a total of 172 articles, of which nine met the inclusion criteria. However, one study used pelvic floor surface electromyography values as the outcome measure and was, therefore, excluded from the analysis. The remaining eight studies (two randomized controlled trials, one pre-post self-controlled study, two case reports, and three prospective studies) were included. The patients included those with chronic prostatitis/CPPS, chronic pelvic pain (CPP), irritable bowel syndrome, perineal pain, and urological CPP syndrome. Table 1 summarizes the characteristics of the included studies and provides a detailed summary of the treatment regimens and results.

**Table 1:** An overview of the protocols and results documented in the studies incorporated within this review.

Study	Design	Type	C/N	Sex	Site	Coil	Protocol	Outcome	AE
Rocco Salvatore Calabrò (79) 2022	Single-center, non-controlled, open-labeled, prospective, pilot	CPP	7/7	F	SMA	MagStim Bistim2 super-rapid stimulator, 70-mm figure-of-eight coil	rTMS-MV paradigm every day, 5 days a week, for 3 weeks. MV (150 Hz, 30 min) with rTMS (10 series of 5 Hz for 10-s pulses with a 40-s interval, 120% of AMT, 500 pulses)	Out of 7 patients, 6 showed at least a 10% improvement in VAS score	Total tolerance Mild headache and neck pain
Jussi Nikkol^a^ (22) 2020	Single-center, non-controlled, pilot	CP/CPPS	11/11	M	M1 (L and R)	MagStim Rapid2 stimulator, figure-of-eight coil	Navigated rTMS over 5 consecutive days, 15 series of 10 Hz for 5-s pulses with a 26-s interval, 110% of RMT, 1,500 pulses	NRS score decreased by 1.2 points (post-treatment), 1.4 points (1 week), and 0.8 points (8 weeks)	Total tolerance Two patients had mild headache
Hasan Hodaj (80) 2020	Single-center, non-controlled, pilot	Perineal pain	18/18	5 M/ 13F	Cz	MagPro stimulator, angled B70 figure-of-eight coil	One session per day for 5 days during 2 consecutive weeks (weeks 1 and 2), followed by 2 sessions in the next week (week 3) for a total of 12 sessions; maintenance therapy was undertaken, consisting of one rTMS session in week 4 and then bimonthly sessions for the next 5 months for a total of 11 sessions. rTMS (40 series of 10 Hz for 5-s pulses with 25-s interval, 80% of RMT, 2,000 pulses)	12 people showed over 30% pain relief.	Total tolerance No serious adverse events
Mauro Cervigni (17) 2018	Multicenter, randomized, double-blind, sham-controlled, crossover	BPS/IC	13/15	F	M1	MagStim Rapid2 stimulator, H-coil,	One group received rTMS-real treatment, and after a 6-week washout period, they received the rTMS-sham (which induced a negligible electric field); another group received the same treatments in inverted order. rTMS-real for 5 days, 30 series of 20 Hz for 2.5-s pulses with a 30-s interval, 110% of RMT, 1,500 pulses	In the real stimulation phase, a significant overall reduction emerged in the VAS score	No serious adverse events
Julien Nizard (61) 2018	Open-label case report	BPS	1/1	F	DLPFC (R and L)	Unspecified	Navigated rTMS of the DLPFC, first on the right hemisphere (one daily session for 5 days, followed by one weekly session for 5 weeks), and then on the left hemisphere (one monthly session for 6 months). 1 Hz delivered continuously, 110% of the MT, 1,200 pulses	Suprapubic pain was reduced (NRS score: 6/10) to completely abolished from the second session	Not mentioned
Tarig Algladi (77) 2015	Single-center, prospective, pilot	IBS	10/10	1 M/ 9F	M1	MagStim Super-Rapid stimulator, 70-mm flat figure-of-eight coil	10 series of 10 Hz for 6-s pulses with a 60-s interval, 80% of RMT, 600 pulses	Anal and rectal pain thresholds increased across all time points after rTMS	Not mentioned
Chloé Melchior (78) 2014	Single-center, randomized, Sham-controlled, double-blind, crossover, pilot	IBS	15/21	10 M/11F	M1	Super-rapid MagStim stimulator, figure-of-eight coil	Real or sham rTMS for 5 days, and after a 2-month washout period, the opposite mode. Neuronavigation rTMS for 5 days, 20 series of 20 Hz, for 5-s pulses with 55-s interval, 80% of RMT, 2,000 pulses	During active or sham rTMS sessions, abdominal pain was significantly reduced	Headaches with mild hand paresis Anxiety
Jean-Marie Louppe (42) 2013	Case report	Perineal pain	2/2	F	M1	Unspecified	One/two sessions of rTMS after implantation of the motor cortex stimulation electrode, rTMS (20 series of 10 Hz For 10-s pulses with 50-s interval, 80% of RMT, 2,000 pulses)	Pain relief was obtained after 1 or 2 days and lasted several days before returning to baseline	Not mentioned

C/N, Number of participants who completed the experiment/Number of cases; AE, adverse effects; CPP, chronic pelvic pain; CP, chronic prostatitis; CPPS, chronic pelvic pain syndrome; BPS, bladder pain syndrome; IC, interstitial cystitis; IBS, inflammatory bowel disease; F, female; M, male; SMA, supplementary motor area; DLPFC, dorsolateral prefrontal cortex; BA4: Brodmann area 4; rTMS, recurrent magnetic transcranial stimulation; MV, muscle vibration; AMT, active motor threshold; RMT, resting motor threshold; MT, motor threshold; VAS, visual analog scale; NRS, numeric rating scale for average and worst pain.

### 3.1 Pain score

**Figure 2:**
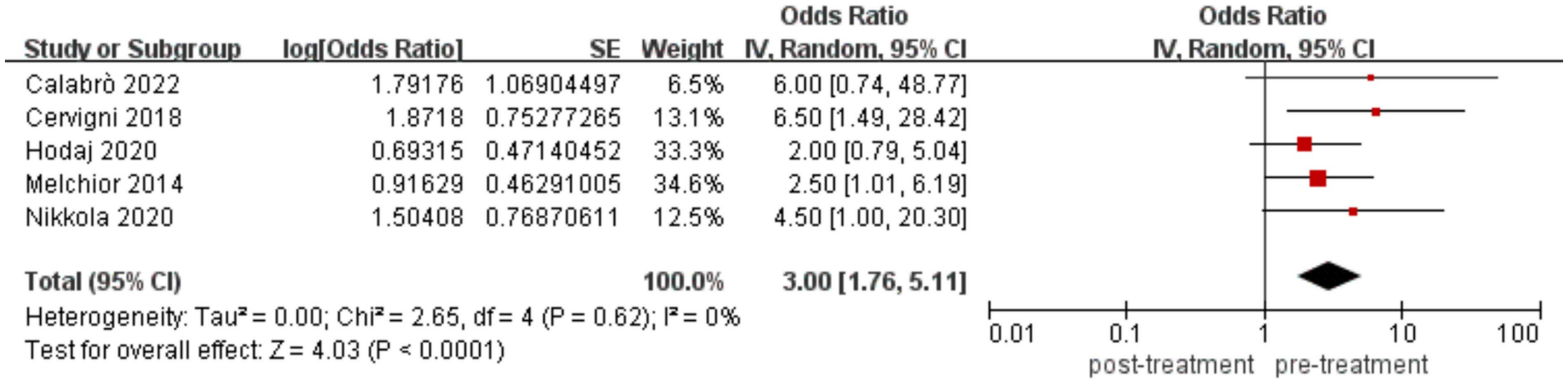
Pain treatment effectiveness rate forest plot.

Analysis of data from the eight studies showed that seven reported a decrease in pain scores, whereas one reported an increase in the pain threshold. The included literature comprised single-arm clinical studies; therefore, the JBI scale was utilized for quality assessment, as shown in Table 2. A total of 85 patients received rTMS, and 77 completed the treatment, with 58 experiencing a decrease in pain scores and 10 showing an increase in the pain threshold. After excluding two case reports and one study with elevated pain thresholds, the effectiveness rates of pain treatment from the remaining five studies were combined using RevMan software and presented in a forest plot, as shown in Figure 2. The visual analog scale (VAS) is the most frequently utilized instrument for pain assessment.

**Table 2:** JBI critical appraisal quality assessment of the case series study.

Study term	Algladi 2015	Calabrò 2022	Cervigni 2018	Hodaj 2020	Louppe 2013	Melchior 2014	Nikkola 2020	Nizard 2018
Were there clear criteria for inclusion in the case series?	Y	Y	Y	Y	N/A	Y	Y	N/A
Was the condition measured in a standard, reliable way for all participants included in the case series?	Y	Y	Y	Y	N/A	Y	Y	N/A
Were valid methods used for identification of the condition for all participants included in the case series?	Y	Y	Y	Y	Y	Y	Y	N
Did the case series have consecutive inclusion of participants?	U	Y	U	Y	N/A	Y	U	N/A
Did the case series have complete inclusion of participants?	U	Y	U	Y	N/A	Y	U	N/A
Was there clear reporting of the demographics of the participants in the study?	U	Y	Y	Y	N/A	Y	Y	N/A
Was there clear reporting of clinical information of the participants?	N	Y	Y	Y	Y	Y	Y	Y
Were the outcomes or follow up results of cases clearly reported?	Y	Y	Y	Y	Y	Y	Y	Y
Was there clear reporting of the presenting site(s)/clinic(s) demographic information?	N	Y	Y	Y	N	Y	Y	N
Was statistical analysis appropriate?	Y	Y	Y	Y	N/A	Y	Y	N/A

Y: Yes; N: No; U: Unclear; N/A: not application.

### 3.2 Transcranial magnetic stimulation frequency

The rTMS frequency ranged from 1 Hz to 20 Hz, with high-frequency stimulation used in seven trials, including 10 Hz in four trials, 20 Hz in two trials, and 5 Hz in one trial. Only one trial used low-frequency (1 Hz) rTMS. The results clearly indicated that high-frequency rTMS had an analgesic effect. Further research is needed to investigate the effectiveness of low-frequency 1 Hz rTMS.

### 3.3 Transcranial magnetic stimulation of brain regions

In the eight trials, the primary target for stimulation was the M1 area (five times), with one describing the target area as Brodmann’s area 4, one trial targeting the Cz point, and the remaining studies targeting the dorsolateral prefrontal cortex (DLPFC) and supplementary motor area (SMA). During the baseline, treatment, and follow-up periods after stimulation, all experiments showed there was modulation of pain in response to rTMS. A comprehensive analysis indicates that transcranial magnetic stimulation of the M1 region of the brain can effectively reduce pain scores in individuals with chronic pelvic pain syndrome. However, further research is necessary to explore its application in the SMA and DLPFC.

## 4 Discussion

CPPS is a pain syndrome that does not involve any distinct pathological alterations. It predominantly involves pelvic-related (pelvic pain and pelvic girdle pain), nerve-related (perineal pain and neuralgia), and anorectal diseases (rectal pain and anal pain), which occur in both sexes; gynecological diseases (pelvic inflammatory disease and pelvic infection), which occur only in females; and prostate-related diseases (chronic prostate pain and prostatitis), which occur only in males. While the etiologies of the pain associated with these conditions may differ, they all impact the pelvic floor region, are characterized by specific symptoms, and lead to chronic and challenging pain management. People have shorter lifespans and spend more of their income on healthcare as a result (20). Numerous social and psychological factors, as well as hypertense pelvic floor muscles (PFMs) and the enhanced sensitivity of peripheral and central nerves, are just some of the multi-faceted causes of CPPS (21). According to a few limited and preliminary findings from recent studies, rTMS shows promise as a treatment for CPPS (22). However, our incomplete understanding of the mechanisms underlying the effects of rTMS and the varying analgesic outcomes of rTMS under different conditions complicate the evidence regarding its effectiveness and safety in individuals with CPPS. Consequently, this discussion focuses on evaluating the efficacy and safety of rTMS by considering factors such as the stimulation device (coil), stimulation parameters, and specific brain regions targeted, all of which can influence treatment outcomes in Table 3.

**Table 3:** Transcranial magnetic stimulation equipment and parameters.

Equipment and parameters		Specificities
Coil	figure-of-eight coil	The figure-of-eight coil is more effective at focusing on superficial areas of the cerebral cortex, but it has a shallower depth of penetration.
	H-coil	H-coils effectively stimulate deep brain structures.
	double-cone coil	double-cone coil Biconical coils stimulate deeper regions of the brain compared to H-coils; however, the risk of optic nerve stimulation is greater
Stimulation frequency	1HZ	Low frequency (≤1 Hz) primarily exhibits a suppression effect.
	5 ~ 20HZ	High frequencies (>1 Hz) are primarily excitatory.
Stimulation of brain areas	M1	Area M1 is associated with central sensitization, altered neuroplasticity, and the inhibitory pathways involved in both ascending and descending conduction.
	SMA	SMA is closely related to the tension in PFM.
	DLPFC	The DLPFC region influences patients’ cognitive experiences and emotional values.

### 4.1 Coils used in TMS

The coil is a crucial factor in determining TMS’s safety and effectiveness, as the outcomes are limited by the device’s hardware (23). The manner in which the electric fields diffuse from several coils positioned on the surface of the brain can have a significant impact on the efficacy and safety of rTMS (24). As such, a careful evaluation of the accuracy and penetration depth of the various stimulation coils is essential in clinical settings. The placement and orientation of the coil are factors that significantly affect the effectiveness of TMS therapy (25). Thus, accurate spatial localization is essential for the effective application of functional magnetic stimulation. In our review of the literature, five studies utilized figure-of-eight coils, one employed an H-coil, while two case reports did not specify the shape of the coil used. In terms of pain relief, one study indicated an elevation in the pain threshold occurred, while the remaining seven all reported noteworthy reductions in pain. Consequently, when selecting a TMS coil, careful evaluation of the benefits and drawbacks of the stimulation focus and depth are important, along with a thorough assessment of the safety considerations associated with TMS.

### 4.2 TMS parameter settings

The precise settings used during the stimulation and the targeted brain regions are intimately associated with the therapeutic effects of rTMS (26). By using various stimulation settings to elicit a variety of neurophysiological reactions, rTMS has the potential to improve recovery (27). TMS acts by coordinating specific neural network patterns of brain activity when specific frequencies are targeted (28). Determining the optimal rTMS frequency for each distinct region of the brain could greatly improve the efficacy of treatments for neurological disorders because different parts of the brain react differently to different frequencies (28, 29). This could pave the way for better brain plasticity adjustments, leading to more successful treatment outcomes. The present study revealed that, in the context of CPPS, most studies used high rTMS frequencies (5–20 Hz), with only one study evaluating the effects of low-frequency (1 Hz) rTMS. Notably, the most commonly utilized frequency was 10 Hz, which was employed in four studies. This insight is completely in line with other studies on the use of rTMS for pelvic floor diseases (18). In a parallel exploration, studies on chronic pain have unveiled comparable findings, highlighting the effectiveness of using high-frequency rTMS at frequencies of between 10 and 20 Hz. Numerous conditions, including migraines, fibromyalgia, peripheral neuropathic pain, and various other types of chronic pain, may be alleviated by this innovative technique (30). Moreover, a narrative analysis of neuropathic pain indicated that the dynamic approach of high-frequency 10 Hz rTMS, targeting the primary motor cortex (M1) and administered over multiple sessions, exhibited a notably enhanced therapeutic impact compared to the more muted low-frequency and one-time TMS techniques (26).

### 4.3 TMS sites

The main motor cortex (M1) located in the precentral gyrus (Brodmann’s area 4) of the human brain is a crucial component of the motor cortex (31). By collaborating with other motor regions, it aids in the regulation of human movement. A multitude of anatomical, functional, and organizational characteristics of the motor cortex are associated with persistent pain (32), and the motor cortex may be impacted by various persistent pain conditions in different ways (33). Studies have revealed that the M1 region is important in affecting our perception of experimental pain as well as in reducing the symptoms of chronic neuropathic pain (34). Pain is influenced by M1 activity, which is linked to pain perception in a number of ways, entailing the inhibition of ascending nociceptive signals in the thalamus and the activation of the descending pain modulation route via the midbrain periaqueductal gray matter (35). One groundbreaking study that looked at the use of rTMS for bladder pain syndrome/interstitial cystitis (BPS/IC) (17) obtained promising results suggesting that rTMS could be a game-changer in easing pelvic pain and related urinary issues. By fine-tuning brain plasticity and reshaping neuronal pathways in the cortex, this innovative treatment may offer new hope for those suffering from these challenging conditions. The SMA is a region of the cerebral cortex that is essential to our capacity for movement and motor coordination (36). When we look at the structure of the brain, the SMA, which is part of Brodmann area 6, is tucked away in the posterior section of the superior frontal gyrus, and is necessary to synchronize and balance the body’s movements (37). The SMA is more important for coordinating and adjusting our motions than the main motor cortex. It is essential for regulating the flow of activity in the primary motor cortex (M1) and for coordinating the performance of motor sequences (36, 38). This regulatory powerhouse is crucial for achieving the smoothness of body movements, optimizing motor function, and ensuring the flawless execution of unilateral brain activity (39). In addition to compensating for injury to M1 and its corticospinal tract fibers, the structural integrity of the SMA and the corticospinal tract nerve fibers emanating from the proximal SMA might affect contralateral motor performance (40). The SMA plays a critical function in pain regulation and the emergence of neuropathic pain by participating in the neural network for pain processing in the cingulate cortex and hippocampal regions (41). The SMA exhibits morphological abnormalities and functional hyperactivity in PFM disorders, including CPPS and urinary incontinence (42–49). Two studies included in this review examined the role of the SMA in regulating the PFM, where excessive tension in the PFM is the primary mechanism contributing to CPP. rTMS has the potential to modulate both the increase and decrease in SMA activity, thereby facilitating either an enhancement or reduction in PFM activity. A prior investigation suggested that low-frequency rTMS aimed at the SMA may enhance PFM activity to address PFM relaxation. On the other hand, by reducing PFM activity, high-frequency rTMS may be very helpful in releasing tension in such muscles (50).

Tucked away in the frontal lobes on both sides of the brain, the DLPFC serves as a hub of neural connections to numerous brain regions (51). Due to its multiple connections to the motor and sensory cortices, this region is crucial for controlling behavior, attention, and influencing cognitive function (52). Research on healthy individuals indicates that the left DLPFC has an inhibitory effect on the ipsilateral M1, whereas the right DLPFC has a comparatively smaller impact on the ipsilateral M1 (53). Additionally, some studies suggest that chronic pain is associated with a reduction in gray matter within the DLPFC (54). Non-invasive brain stimulation techniques have the power to reshape the structure and operation of the DLPFC. Using rTMS to stimulate the DLPFC can change how we perceive pain, as it not only modifies the emotional significance we attach to pain but also counteracts the shifts in motor cortex excitability that painful experiences can trigger. In the end, this helps to relieve discomfort (55). By interacting with other regulatory systems, such as the opioid pathways and our cognitive and emotional reactions, this alteration can also alter how we feel pain (56). In addition, rTMS stimulation of the DLPFC can alter the activity of the emotional regulation network by affecting the functional connectivity of the amygdala, thereby modulating pain perception (57–60). A study using rTMS of the DLPFC in patients with bladder pain syndrome (BPS) found that low-frequency TMS of the right DLPFC improved the symptoms. In addition, treatment outcomes were enhanced and depression symptoms were reduced when the left DLPFC was activated (61). Based on these studies, we can infer that the M1 area of the brain is associated with central sensitization, alterations in neural plasticity, and the inhibitory pathways of both ascending and descending transmission. The SMA is closely linked to PFM tension, and the DLPFC area mutually influences the cognitive experience and emotional value of the patients.

Our preference for using our left or right hand is strongly connected with our brain’s reaction to TMS (62, 63). This is because unique structural and functional differences in our brains lead to varying reactions to pain. For example, a study discovered a connection between our sensitivity to pain and how we utilize our dominant and non-dominant hands (64). Moreover, a plethora of research suggests that left-handed individuals experience different degrees of pain sensitivity than their right-handed counterparts. This might be connected to their non-dominant hand’s increased sensitivity to pain, which results in a decreased degree of total lateralization (65). Studies focusing on infants have revealed that their hand dominance could play a role in how they experience pain. This connection is explained by the activation of the prefrontal lobe in reaction to negative experiences (66). Human handedness is associated with the integrity of the white matter pathways in the brain (67). Variations in white matter connectivity may provide insight into the effects of TMS on brain activity and cognitive function (63). Consequently, when evaluating TMS’s efficacy in modulating brain function, the influence of handedness is an important consideration.

### 4.4 TMS security

In TMS, the pulsed electromagnetic field generated by the coil can produce significant noise that might disrupt and obscure the desired effects of the treatment. Tinnitus, hearing loss, and a lowered tolerance for sound are among some of the safety concerns (68). The risk associated with sound pulses should be considered while selecting coils, even if it can be mitigated by the patients donning the appropriate protection gear (69, 70). Since the issuance of safety guidelines on TMS by the International Federation of Clinical Neurophysiology (IFCN) in 1998, the incidence of TMS/rTMS-induced seizures has been notably low. Seizures caused by rTMS are also unlikely to recur after the operation, unless the patient has a history of epilepsy (23). The moderate side effects of TMS, which are rather common, may depend on the participants’ initial expectations or anxiety levels (71). Recent evaluations have demonstrated that TMS is a safe and effective therapy option for a range of patient populations, including children, teens, adults, peripartum women, and older adults (72–75). The studies assessed in this review indicated that adherence to the recommended stimulation frequency and intensity parameters generally results in well-tolerated responses among individuals, with only minor adverse events and no instances of severe reactions documented. These findings demonstrate the safety of TMS treatment for CPPS, which is consistent with earlier safety assessments of TMS treatment for chronic neuropathic pain (76).

### 4.5 Limitations

The primary study limitations identified include the heterogeneity across various studies, the incomplete assessment of the overall extent of pain relief, discrepancies in the targeted stimulation sites, and inconsistencies in the parameters used for stimulation.

## 5 Conclusion

Evaluations of the safety of rTMS suggest that it is a promising non-invasive approach for managing CPPS. The reviewed studies indicated that targeting the M1 motor area with rTMS can enhance brain plasticity, regional brain connectivity, and brain–spinal-cord pathways, and improve the effectiveness of pain mitigation (17, 77, 78). However, when CPPS results from elevated PFM tension, stimulating the SMA with rTMS frequently yields better results (48, 79). Lastly, in patients with concurrent mental diseases, stimulation of the DLPFC region can help regulate mood and significantly lessen symptoms (61). Another important factor to consider is whether someone is left-handed or right-handed.

Overall, this review summarizes the analgesic effect of rTMS on CPPS. Although TMS is a safe and promising technique that can reduce long-term refractory pain, its clinical application is still hindered by the variability in stimulation parameters and inclusion criteria, diverse etiologies, varied outcome assessment methods, and limited randomization of studies, all of which lead to a high risk of bias. Therefore, additional research efforts are necessary to determine the most effective stimulation protocols and to standardize all pertinent parameters. This will contribute to improving the long-term effectiveness of rTMS as a noninvasive treatment modality for the management of CPPS.

## Data Availability

The original contributions presented in the study are included in the article/Supplementary material, further inquiries can be directed to the corresponding author.
