# Supplementary figures and images for: Clinical application of repetitive transcranial magnetic stimulation in the treatment of chronic pelvic pain syndrome: a scoping review
Source: Front Neurol. 2025 Feb 26;16:1499133. doi: 10.3389/fneur.2025.1499133 (PMC11905899; doi:10.3389/fneur.2025.1499133)

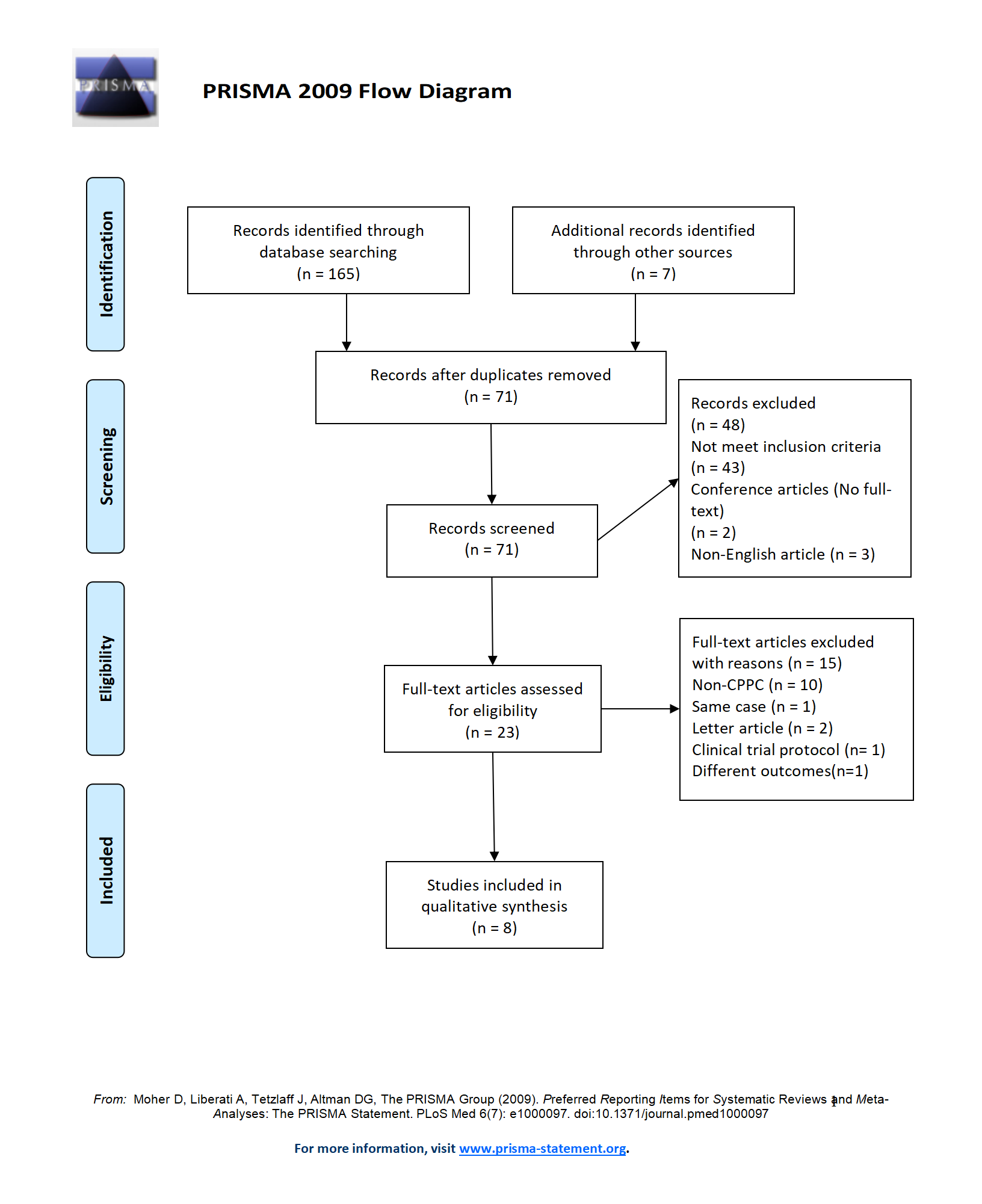

Supplement: Supplementary file 1 [file Image_1.png]
